# Efficacy and safety of heart rate control with esmolol on the incidence and duration of organ failure in predicted severe acute pancreatitis: protocol of a multicenter, open-label, randomized controlled trial

**DOI:** 10.3389/fmed.2025.1642721

**Published:** 2025-07-30

**Authors:** Mingfeng Huang, Rong Wei, Lu Ke, Weijian Li, Jian Song, Haibin Ni, Xiaofei Huang, Yun Sun, Lu Fu, Yanhua Li, Dong Zhang, Bin Han, Jing Zhang, Yingying Hu, Chong Zhang, Zhongyan Sheng, Wenwen Feng, Lin Gao, Wenjian Mao, Yuxiu Liu, Bo Ye, Zhihui Tong, Weiqin Li

**Affiliations:** ^1^Department of Critical Care Medicine, Jinling Hospital, Affiliated Hospital of Medical School, Nanjing University, Nanjing, China; ^2^Jinling Clinical Medical College, Nanjing Medical University, Nanjing, China; ^3^The Affiliated Hospital of Guizhou Medical University, Guiyang, Guizhou, China; ^4^Affiliated Hospital of Integrated Traditional Chinese and Western Medicine, Nanjing University of Chinese Medicine, Nanjing, China; ^5^The First Department of Critical Care Medicine, The Second Affiliated Hospital of Anhui Medical University, Hefei, Anhui, China; ^6^Department of Critical Care Medicine, The First Hospital of Jilin University, Changchun, China; ^7^Department of Emergency, The First People's Hospital Yunnan Province, The Affiliated Hospital of Kunming University of Science and Technology, Kunming, Yunnan, China; ^8^The First Affiliated Hospital of Henan University of Science & Technology, Luoyang, Henan, China; ^9^Department of Critical Care Medicine, Qianxinan People's Hospital, Qianxinan, Guizhou, China; ^10^Department of Biostatistics, School of Public Health, Nanjing Medical University, Nanjing, Jiangsu, China

**Keywords:** acute pancreatitis, *β*-blockers, esmolol, heart rate, organ function

## Abstract

**Introduction:**

Overactivation of the sympathetic nerve system can lead to a sustained increase in heart rate, which may impair blood perfusion and organ function. Previous studies have demonstrated that the use of *β*-blockers like esmolol can reduce heart rate, thereby improving clinical outcomes in patients with septic shock. For acute pancreatitis (AP), which shares a similar inflammatory pathophysiology with sepsis, previous experimental and observational studies showed significant sympathetic excitation during the acute phase, and the use of *β*-blockers might be clinically beneficial. This study aims to test the hypothesis that early intravenous esmolol administration to control heart rate will improve the incidence and duration of organ failure in patients with predicted severe acute pancreatitis (pSAP).

**Methods:**

This is an investigator-initiated, multicenter, open-label, randomized controlled trial. All patients with pSAP who still exhibit elevated heart rate (≥110 bpm) after 6 h of adequate intravenous fluid resuscitation within the first 72 h of symptom onset will be screened for eligibility. A total of 146 participants will be randomized to receive either esmolol or standard care. Patients in the esmolol group will receive a continuous esmolol infusion to maintain a heart rate between 80 and 94 beats per minute (bpm) within the first 96 h of randomization. The primary endpoint is organ failure free and alive days (OFFDs) to day 14 after trial entry. Secondary endpoints are comprised of both process and clinical measures, including heart rate variability, the proportion of patients’ heart rate recovered to <95 bpm, changes in plasma interleukin-6 and C-reactive protein between day 1 and day 5, in hospital and 90-day mortality, new-onset organ failure, free and alive days to day 30 for intensive care admission, and requirement of mechanical ventilation, vasopressor use, and renal replacement therapy.

**Discussion:**

This study will provide top-class evidence concerning the effects of heart rate control with a classic *β*-blocker on the incidence and duration of organ failure in patients with pSAP and increased heart rate.

**Ethics and dissemination:**

This study has been approved by the ethics committee of Jinling Hospital, Nanjing University (2022DZKY-076-02) and all participating sites. Results will be disseminated through peer-reviewed journals and scientific conferences.

**Trial registration:**

Identifier, ChiCTR2400080160.

## Introduction

Acute pancreatitis (AP) is a systemic inflammatory disease with significant morbidity, and about 20% of patients, who have more intense inflammation, develop severe AP (SAP) (1, 2). The inflammatory response, combined with intense stress and pain, leads to elevated sympathetic activation in SAP patients (3). In turn, the persistent excitation of the sympathetic nervous system can reciprocally intensify inflammation, triggering the release of inflammatory mediators and perpetuating a vicious cycle (4).

Given that sympathetic activation is a hallmark of inflammatory diseases (5, 6), numerous efforts have been undertaken to modulate its activity in the hope of improving clinical outcomes (7, 8). Recent studies suggest that modulating sympathetic nerve activity using *β*-1 adrenergic blockers may offer therapeutic benefits in sepsis by attenuating excessive inflammatory responses and organ failure (6, 9). Esmolol, a short-acting intravenous *β*-blocker, was previously used to treat inflammatory conditions like sepsis (10, 11). It works by inhibiting the effects of adrenaline on sympathetic overactivation, thereby reducing heart rate to improve tissue perfusion and suppressing inflammatory mediators (12).

A phase II clinical trial involving 154 patients with septic shock demonstrated that esmolol could reduce heart rate without affecting mean arterial pressure (13). Notably, esmolol significantly reduced 28-day mortality (49.4% in the esmolol group vs. 80.5% in the control group, *p* < 0.001) (13). Another study in 2015 found that combining milrinone and esmolol improved cardiac function and 28-day survival in patients with severe sepsis (14). Recently, two large-scale trials were published testing the effects of a new ultra-short-acting *β*-blocker, landiolol, in patients with septic shock and persistent tachycardia [The Landi-SEP (15) and STRESS-L (16)]. The former found that landiolol was effective in reducing and maintaining heart rate without increasing vasopressor requirements after 24 h. However, the latter was halted prematurely after enrolling only 37% of the planned participants. The decision was made due to the lack of evidence suggesting that landiolol could improve organ function, coupled with indications of potential mortality harm. The most likely explanation, as the authors suggested, may be compromised cardiac output in patients with already existing shock.

From the pathophysiological perspective, elevated heart rate and cardiac output typically occur during the acute phase of AP, primarily mediated through systemic inflammatory activation, which is associated with excitation of the sympathetic nervous system (17). Our previous study showed that elevated sympathetic activity was strongly associated with a poor prognosis (3). Interventions such as electroacupuncture, which stimulate the vagus nerve and, in turn, reduce sympathetic nervous system activity, have been shown to reduce pancreatic injury in AP mice (18). However, based on observations in sepsis trials, it is also possible that the use of *β*-blockers may reduce cardiac output, thereby compromising tissue perfusion. Taken together, due to the lack of controlled trials, the efficacy and safety of early heart rate control with *β*-1 adrenergic blockers on outcomes in AP patients, particularly those at high risk of SAP, remains unclear. This trial aims to evaluate whether early intravenous esmolol administration to control heart rate can reduce the incidence and duration of organ failure in patients with predicted severe acute pancreatitis (pSAP).

## Methods and analysis

### Aim and objectives

The *β*-Blocker esmolol on the evolutioN of organ faIlure in aCute pancrEatitis (BeNICE) trial aims to assess the effect of early heart rate control with intravenous esmolol on the incidence and duration of organ failure in patients with pSAP.

### Study design

This investigator-initiated, multicenter, open-label, randomized controlled trial will be conducted among 8 to 10 participating sites over 12–24 months. The trial is registered at the Chinese Clinical Trials Registry (ChiCTR2400080160).

### Trial committees

The Trial Management Committee (TMC), comprising primary investigators, co-investigators, and members of the Chinese Acute Pancreatitis Clinical Trials Group (CAPCTG), will oversee the trial’s daily operations. An expert clinical panel from CAPCTG will provide governance and audit the study. A writing and publication committee will draft and submit the manuscript to academic journals and determine authorship.

### Study population

Adult patients diagnosed with AP, admitted to participating sites, will be assessed for eligibility. After obtaining informed consent, eligible participants will be randomized.

### Eligibility criteria

The inclusion and exclusion criteria are as follows:

*Inclusion criteria:*

1. Diagnosis of acute pancreatitis (2): symptoms and signs of AP based on abdominal pain, serum amylase at least three times the upper limit of the normal range, and/or characteristic findings on computed tomography;
2. Within 72 h of symptom onset;
3. Predicted severe acute pancreatitis, based on APACHE-II score≥8 or CRP > 150 mg/L (19);
4. Heart rate ≥110 bpm sustained for ≥10 min after at least 6 h of adequate intravenous fluid resuscitation;
5. Age ≥18 years;
6. Written informed consent from the patient or next of kin;

The treating physician should make sure that adequate fluid resuscitation has been delivered before initiation of the trial intervention. Adequate fluid resuscitation should be achieved using repeated fluid challenges based on current guidelines (20). Potential targets include any or all of the following based on the clinical setting of the study site: (1) Mean arterial pressure between 65–85 mmHg, (2) Good peripheral perfusion on clinical examination, e.g., urinary output > 0.5 mL/kg/h, blood lactate < 2 mmol/L, (3) Other measures of cardiac output/flow, e.g., cardiac output, stroke volume variability, (4) Central venous pressure ≥8 mmHg, (5) Biochemical targets of hematocrit 35–44%.

*Exclusion criteria:*

1. Patients who had received *β*-blocker therapy before enrollment during the index admission;
2. Patients with chronic cardiac dysfunction (New York Heart Association Classification III or above);
3. Pregnancy;
4. Active bronchial asthma or history of asthma;
5. Severe chronic obstructive pulmonary disease requiring home oxygen therapy;
6. Predicted death within 24 h;
7. Patients with contraindications to *β*-blockers (e.g., severe bradycardia, advanced heart block, cardiogenic shock, untreated pheochromocytoma).

### Randomization and blinding methods

Permuted block randomization (variable block size = 4 or 6), stratified by site, will be performed using a computer-generated list via the Interactive Web Response System (IWRS). The study is open-label, but laboratory personnel and data analysts will be blinded to the allocation sequence.

### Sample size, centers, and recruitment

Based on previous large studies [the CLEVER-AP (21) and the PERFORM (22)] conducted by the CAPCTG, the mean organ failure free and alive days (OFFDs) to 14 days in patients with pSAP was estimated as 7.3 (SD 4.1). Both of the two studies were large-scale ones conducted across China in similar population. The former aimed to compare the effect of a balanced solution versus normal saline fluid therapy in patients with pSAP. The latter was a long-running prospective cohort study collecting patients with hypertriglyceridemia-associated acute pancreatitis. Per the standard formula calculated with PASS (ver.15.0; NCSS, LLC),^1^ a sample size of 66 patients per group provides 80% power to detect a two-day difference in OFFDs between groups, which is thought to be the minimum clinically meaningful treatment effect. After accounting for a 10% loss of follow-up, 146 patients (73 per group) are planned to be randomized.

The first patient was recruited on Feb 8th, 2024, and the planned finishing date is Dec 31st, 2025. The follow-up will be finished after the 90-day follow-up of the last recruited patient is completed.

### Study interventions

The study protocol only determines whether or not to use esmolol.

- Group 1: Esmolol group

Patients will receive esmolol infusion within 1 h of randomization, starting at 25 mg/h, titrated to maintain a heart rate of 80–94 bpm, with a maximum dose of 0.2 mg/kg/min. The infusion will continue for 96 h unless: (1) the heart rate decreases below 80 bpm and re-evaluation needs to be performed after 20 min; (2) systolic blood pressure falls below 90 mmHg; (3) severe arrhythmias develop; or (4) the clinician deems it necessary based on the patient’s condition.

Meanwhile, we will implement stringent measures to mitigate the risk of low cardiac output in the study patients, including: (1) adequate fluid resuscitation prior to esmolol initiation; (2) patients assigned to the esmolol group had their blood pressure monitored every 15 min. and (3) cardiac output monitoring when necessary, using techniques like echocardiography, invasive hemodynamic monitoring, etc., as judged by the treating physicians.

- Group 2: Standard care group

Patients will receive standard care without a placebo at the discretion of the treating physicians.

### Management of acute pancreatitis

Apart from the trial intervention, all the study patients will be managed by the local clinical team at each participating site based on international guidelines (20, 23), including adequate fluid resuscitation, early enteral nutrition (start within 48 h after admission), routine medical treatment (antibiotics and sedatives as needed, organ support like mechanical ventilation, renal replacement treatment (RRT), and vasopressors commenced at the discretion of the treating team), etc. Infected pancreatic necrosis will be managed using a step-up approach, either surgically or endoscopically, depending on the technical preference of the local team (20, 24).

### Endpoints

#### Primary endpoint

The primary endpoint is organ failure free and alive days (OFFDs) to 14 days of enrollment, defined as the number of days alive without failure of the respiratory, renal, or cardiovascular system (25). An individual sequential organ failure assessment (SOFA) score of two or more was defined as organ failure (26). Patients discharged from the hospital prior to day 14 were considered alive and free from organ failure from the day of discharge onward. For patients who died before day 14, OFFDs were assigned a value of zero. If patients are readmitted to the index study site with recurrent organ failure within 14 days, only the last period alive and free of organ failure will be counted.

#### Secondary endpoints

Part I: process endpoints

1. Proportion of patients’ average heart rate recovered to < 95 bpm at different time periods after enrollment (24 h, 48 h, 72 h, and 96 h after enrollment). This assessment was derived from continuous heart rate variability (HRV) monitoring, with meticulous exclusion of factors including sputum suction procedures, patient handling, and other medical interventions that could potentially influence heart rate.
2. HRV within 24 h, 48 h, 72 h, and 96 h after enrollment
3. Changes in plasma interleukin-6 between day 1 and day 5
4. Changes in plasma C-reactive protein between day 1 and day 5

*Part II: Patient centered endpoints*

1. In hospital and 90-day mortality
2. Proportion of intensive care unit (ICU) admission
3. Changes in pancreatitis activity scoring system (PASS) scores from day 1 to day 7 after enrollment
4. Incidence of bleeding requiring intervention
5. Incidence of gastrointestinal perforation or fistula
6. Incidence of pancreatic fistula
7. Proportion of patients whose enteral nutrition meets the target nutrient intake on day 7 and day 14
8. New-onset organ failure as judged by SOFA score
9. New-onset mechanical ventilation
10. New-onset RRT
11. New-onset vasoactive drug therapy
12. ICU free and alive days to day 14
13. Ventilator free and alive days to day 14
14. Vasopressor free and alive days to day 14
15. RRT free and alive days to day 14
16. Requirement of minimally invasive drainage
17. Requirement of open surgery
18. Length of hospital stay
19. Expense of the index hospitalization
20. Incidence of infected pancreatic necrosis (IPN)

### Monitored parameters and data collection

**Figure 1:**
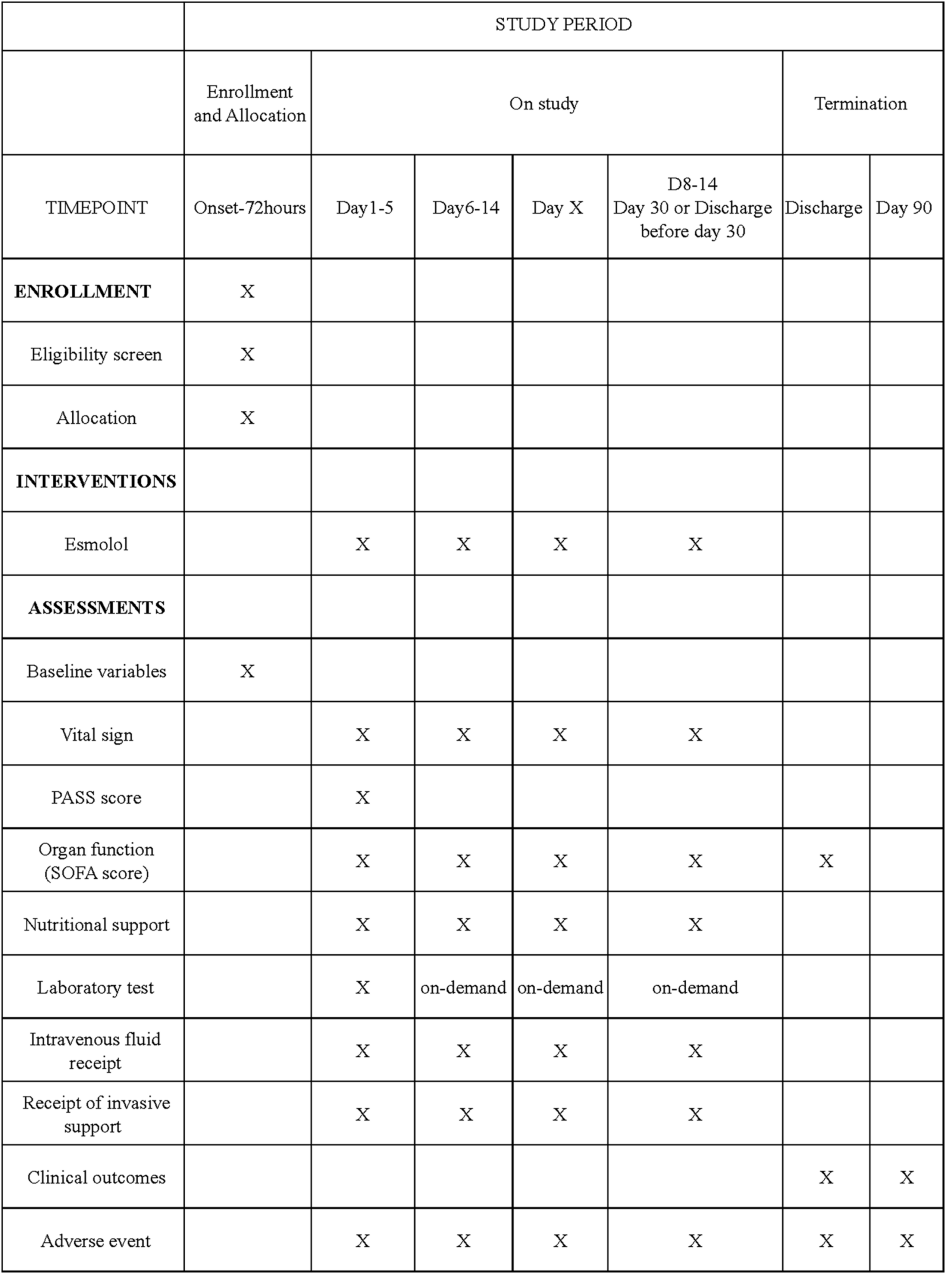
Schedule of enrollment, interventions, and assessments. PASS denotes pancreatitis activity scoring system. SOFA denotes sequential organ failure assessment.

The coordinating center of the CAPCTG will be responsible for data safety, privacy, and quality of the study conduct. Data collection and storage will use a web-based electronic database (Unimed Scientific, Wuxi, China). All data are input by the nominated investigator at each participating site. Training for data entry was arranged by the CAPCTG coordinating center before the study commencement. For the secondary endpoints requiring subjective assessments, all site investigators underwent centralized training on outcome assessment. The data required to be collected during different phases are shown in Figure 1.

### Statistical analysis

The reporting and presentation of this trial will follow the CONSORT guidelines (27). Primary analyses will be based on the intention-to-treat (ITT) population, and secondary supportive analyses will be done on the Per-protocol (PP) population. The safety analysis will be performed on the safety population (the same as the ITT population). Missing primary outcomes will be assumed to be missing at random (MAR) and thus will be ‘ignored’ in the primary analysis. However, if greater than 5% of all primary outcomes that should be available for analysis are missing, a series of sensitivity analyses after imputing the missing primary outcome will be undertaken in addition to the primary analysis. The ITT population consists of all randomized subjects, regardless of whether they are ineligible, prematurely discontinue treatment, or are otherwise protocol violators/deviators. The PP population is a subset of the ITT population that excludes those with major protocol deviations. Major protocol deviations will be defined in the statistical analysis plan.

The normality of continuous variables was examined using skewness and kurtosis. Continuous data were expressed as mean±standard deviation or median (interquartile range). Categorical data were expressed as numbers and percentages. A generalized linear mixed model that accounts for the random effect of different participating sites, and adjusted for several clinically important covariates will be employed to compare group differences in the primary outcome. The detailed analysis strategies for secondary outcomes and subgroup analyses by APACHE II score ≥8 (yes and no), CRP > 150 mg/L (yes and no), etiologies of AP (hypertriglyceridemia and non-hypertriglyceridemia), the presence of organ failure (yes and no), presence of shock (yes and no), and time interval between abdominal pain onset and enrollment ≤ 24 h (yes and no) will be included in the statistical analysis plan. Statistical tests will be two-sided, and *p* values <0.05 will be deemed statistically significant.

### Adverse events

All adverse events (AEs) occurring from the time of randomization to the final follow-up visit on day 90 will be reported to the CAPCTG coordinating center. AEs are defined as any untoward medical occurrence in a patient who received an investigational intervention. A serious adverse event(SAE) is defined as any serious medical event that causes death, life-threatening conditions, prolonged hospital stay, persistent disability or dysfunction, or other unpredictable serious medical events. The causal relationship between the AEs and the study drug will be classified as probable, possible, or unlikely (28). All AEs were systematically evaluated by the investigators to determine their potential relationship to the study intervention. In particular, bradycardia (heart rate <50 bpm) with hemodynamic compromise requiring intervention, significant hypotension requiring intervention, and arrhythmia with hemodynamic compromise requiring intervention would be recorded, and the association with treatment will be assessed.

An independent data safety and monitoring board (DSMB) (consisting of a surgeon, an intensivist, and a statistician) will oversee all aspects of patient safety based on reported AEs. No formal interim analysis was planned due to the limited sample size of the trial. The DSMB will review the AEs every 6 months, and it can call for a meeting for reported SAEs whenever deemed necessary during the trial. AEs will be reported in a uniform format through the electronic data capture system.

### Trial status

The BeNICE trial is currently recruiting participants. This trial will be conducted between Feb 1st, 2024, to Dec 31st, 2025. The first patient was recruited on Feb 8th, 2024. As of December 2024, there have been 73 patients recruited, approximately 50% of its target. Recruitment is expected to be completed in December 2025.

## Discussion

Although numerous studies have explored the impact of *β*-blockers on the prognosis of critically ill patients, particularly those with septic shock, the findings have been inconsistent and, at times, contradictory. As a distinct subgroup of critically ill patients, it remains uncertain whether early heart rate control using *β*-blockers confers clinical benefits in patients with severe acute pancreatitis. The BeNICE trial aims to provide the first piece of top-class evidence regarding the effect of esmolol on the incidence and duration of organ failure in a cohort of pSAP. The results of this study are expected to have significant clinical implications, offering conclusive insights into this therapeutic approach.

The incidence and duration of organ failure are the key determinants of severity in patients with AP. The primary outcome measure, organ failure free and alive days (OFFDs) to day 14, is closely associated with survival and will allow for the evaluation of esmolol’s impact on organ failure compared to standard care (13, 29). Additionally, the analysis of individual SOFA scores for respiratory, renal, and cardiovascular systems will help determine whether improvements in tissue perfusion, are the sole mechanism driving differences between the groups. Furthermore, the collection and analysis of HRV will provide deeper insights into the role of the sympathetic nervous system in the pathophysiology of AP. Apart from early organ failure, we also look into the latter complications, like IPN. In the acute phase of pancreatitis, associated with sympathetic overactivation, a cascade of inflammatory cytokines is released (30), which potentially leads to “immune paralysis” due to immune exhaustion (31). Esmolol could act to modulate sympathetic overactivity, disrupting the neuroendocrine-immune crosstalk, which may potentially prevent consequent immunosuppression and the development of IPN (3).

Despite the importance of the sympathetic nerve system, data regarding elevated heart rate [an important marker of sympathetic overactivity (32)] and clinical outcomes in acute pancreatitis are lacking. During the design of the trial, the TMC discussed the heart rate threshold that would be used in the eligibility criteria, referencing data from patients with sepsis, a condition sharing similar pathophysiology with acute pancreatitis. The TMC agreed that using the common criterion of a heart rate greater than 100 bpm may involve a significant number of low-risk patients, whereas a meta-analysis of esmolol in septic shock suggested a heart rate of 110 bpm or greater as a potential threshold for therapeutic benefit (33). This threshold was also supported by other studies conducted in sepsis patients (34, 35). Moreover, requiring the elevation (>110 bpm) to be sustained for at least 10 min helps exclude patients experiencing only transient, potentially artifact-driven tachycardia (e.g., due to brief procedure-related distress or movement) rather than sustained physiological stress relevant to their clinical condition.

The BeNICE trial is sponsored by Jinling Hospital of Nanjing University, a nationwide referral center for AP. The trial is conducted by the Chinese Acute Pancreatitis Clinical Trials Group (CAPCTG, capctg.medbit.cn), which has extensive experience in implementing large-scale trials (36, 37).

### Strengths and limitations

#### Strengths

1. This multicenter, open-label, randomized controlled trial provides top-class evidence concerning the impact of early heart rate control with intravenous esmolol on the incidence and duration of organ failure, a critical determinant of the severity of AP.
2. This trial enrolled patients meeting the criteria for predicted severe acute pancreatitis, a well-defined population consistently employed in multiple large-scale randomized trials (19, 21, 38–40). This design enhanced both the generalizability and comparability of our findings in this high-risk cohort.
3. Though the trial is of pragmatic design, a series of objective measures were taken to ensure the safety of the study patients. Similar measures were successfully implemented in a recently published trial testing esmolol in hyperkinetic septic shock patients (41).
4. An independent Data Safety Monitoring Board will review the data to ensure participant safety throughout the trial.

#### Limitations

1. Patients with mild acute pancreatitis, who might also benefit from esmolol, are excluded from this study.
2. The study’s sample size is relatively small and may lack sufficient power to detect differences in key patient-centered outcomes such as mortality. Since this is the first trial investigating the efficacy of *β*-blockers in acute pancreatitis, our sample size estimation may be overoptimistic.
3. The study is not double blind since the dosing of esmolol varies according to a physiological endpoint (target heart rate of 80–94 bpm), a placebo would be ineffective, and the intervention impossible to blind. Moreover, the open-label design may introduce performance bias in subjective endpoint assessments.

## Trial status

The BeNICE trial will be conducted between Feb 1st, 2024, to Dec 31st, 2025. The first patient was recruited on Feb 8th, 2024. As of December 2024, there have been 73 patients recruited, approximately 50% of its target.

## Footnotes

^1^ https://www.ncss.com/software/pass/
